# Magnetite Nanostructures as Novel Strategies for Anti-Infectious Therapy

**DOI:** 10.3390/molecules190812710

**Published:** 2014-08-20

**Authors:** Ioannis Liakos, Alexandru Mihai Grumezescu, Alina Maria Holban

**Affiliations:** 1 Nanophysics Department, Istituto Italiano di Tecnologia (IIT), via Morego 30, Genova 16163, Italy; 2 Department of Science and Engineering of Oxide Materials and Nanomaterials, Faculty of Applied Chemistry and Materials Science, University Politehnica of Bucharest, Polizu Street No. 1-7, Bucharest 011061, Romania; 3 Microbiology and Immunology Department, Faculty of Biology, University of Bucharest, Aleea Portocalelor, No. 1-3, Bucharest 060101, Romania

**Keywords:** infectious diseases, antimicrobials, antibiotics, magnetic nanoparticles, drug delivery

## Abstract

This review highlights the current situation of antimicrobial resistance and the use of magnetic nanoparticles (MNPs) in developing novel routes for fighting infectious diseases. The most important two directions developed recently are: (i) improved delivery of antimicrobial compounds based on a drastic decrease of the minimal inhibition concentration (MIC) of the drug used independently; and (ii) inhibition of microbial attachment and biofilm development on coated medical surfaces. These new directions represent promising alternatives in the development of new strategies to eradicate and prevent microbial infections that involve resistant and biofilm-embedded bacteria. Recent promising applications of MNPs, as the development of delivery nanocarriers and improved nanovehicles for the therapy of different diseases are discussed, together with the mechanisms of microbial inhibition.

## 1. Introduction

Antimicrobial resistance (AMR) is not a recent phenomenon, but in the last few decades bacteria, fungi and yeasts have developed considerable resistance against many of the traditional and modern synthetic drugs. Nowadays the movement of people and goods is much easier than in the past and therefore infectious diseases that have developed in a specific place may easily be transferred worldwide. The uncontrolled use of antibiotics during animal breeding has increased the AMR towards medicines and has made the treatment of infections even more complicated. The factors and mechanisms of how AMR occurs have been identified, but more research is still needed in this field, especially in order to create new antibiotics that will be more effective against the versatile and easily adaptive pathogenic microorganisms. It is well known that AMR has led to high rates of morbidity and mortality among the population, decreased productivity, economic losses, worsening of health related living conditions, psychological implications and increased diagnosis and treatment costs [1].

**Figure 1:**
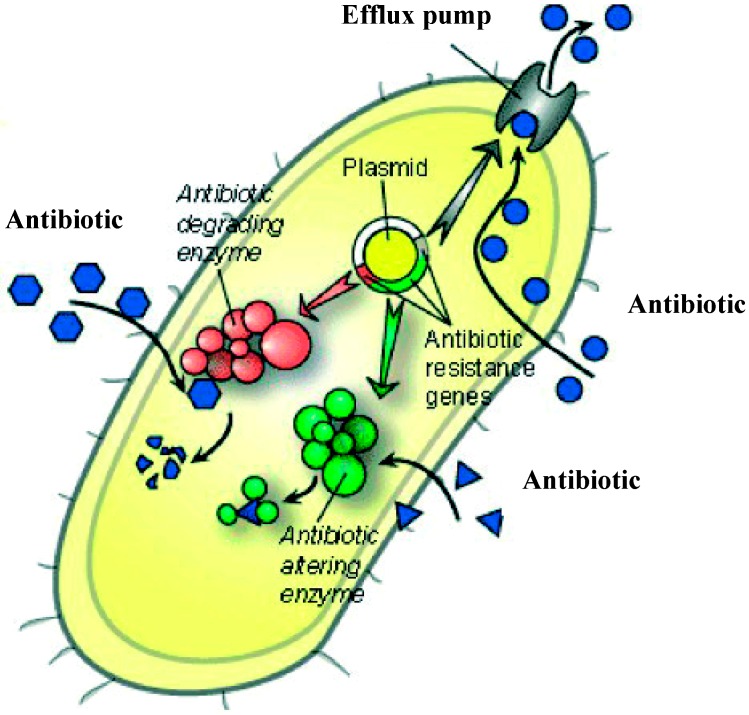
The main mechanisms of AMR: overproduction of efflux pumps, antibiotic modification and antibiotic degradation (reproduced from [2] with permission).

The use of antibacterial drugs in the last decades has caused genetic and biochemical alterations of bacteria to ensure the survival and multiplication of the latter. Resistance can be explained as a mechanism of genetic events causing biochemical changes in their genome such as point mutations and gene amplifications. Additionally, usage of antibiotics has enhanced the accumulation of genetic elements coding for resistance than can be transferred between microbes, creating multi-resistant clones. In antimicrobial resistant bacteria the targeted molecules are structurally altered to prevent antibiotic binding by the following mechanisms: blockage of antibiotics from cell entry, inactivation of antibiotics such as from enzymatic degradation and exit of antibiotics from the cell before binding to any site, through powerful efflux pumps [2]. A general mechanism of AMR is presented in Figure 1.

The effect on morbidity and mortality of infectious diseases is very significant among the population. For example, in 2007 and only in the EU Member states, including Iceland and Norway, around 25,000 people died due to the most common bacteria such as *Staphylococcus aureus*, *Enterococcus faecium*, *Streptococcus pneumoniae*, *Escherichia coli*, *Klebsiella pneumoniae* and *Pseudomonas aeruginosa*. In the same time the morbidity from these bacteria reached almost 400,000 people. The results (Table 1) were obtained from a joint study by the European Centre for Disease Prevention and Control (ECDC) and the European Medicines Agency (EMEA) [3]. Additionally, laboratory reports have shown that bacterial-caused pneumonia kills about 1.9 million children worldwide every year [4]. These data show how current AMR causes problems, highlighting the needed for action to better treat bacterial infections and/or to develop new drugs that can be more effective than the currently used antibiotics.

**Table 1:** Estimated 2007 morbidity and mortality due to selected antibiotic-resistance bacteria in EU Member States, Iceland and Norway (reproduced from [3] with permission).

Antibiotic-Resistant Bacteria	Number of Cases of Infection *	Number of Extra Deaths	Number of Extra Hospital Days
**Antibiotic-resistant Gram-positive bacteria**			
Methicillin-resistant *Staphylococcus aureus* (MRSA)	171,200 (12%)	5400 (37%)	1,050,000 (16%)
Vancomycin-resistant *Enterococcus faecium*	18,100 (9%)	1500 (28%)	111,000 (22%)
Penicillin-resistant *Streptococcus pneumoniae*	3500 (27%)	Not calculable	Not calculable
Sub-total	192,800 (12%)	6900 (35%)	1,161,000 (16%)
**Antibiotic-resistant Gram-negative bacteria**			
Third-generation cephalosporin-resistant *Escherichia coli*	32,500 (27%)	5100 (52%)	358,000 (27%)
Third-generation cephalosporin-resistant *Klebsiella pneumoniae*	18,900 (27%)	2900 (52%)	208,000 (27%)
Carbapenem-resistant *Pseudomonas aeruginosae*	141,900 (3%)	10,200 (7%)	809,000 (3%)
Sub-total	193,300 (9%)	18,200 (27%)	1,375,000 (13%)
**Total**	**386,100 (11%)**	**25,100 (29%)**	**2,536,000 (14%)**

* Bloodstream, lower respiratory tract, skin & soft tissue and urinary tract infections. Values in parentheses correspond to bloodstream infections only.

In 2001 the WHO (World Health Organization) global strategy for the control of AMR [5] provided a framework of interventions that should take place to reduce infectious diseases, including the reduction of spread infections, improvements in the use of and access to antimicrobials, the strengthening of health systems and health legislation and encouragement of the development of new drugs. From the 1930s until now some new classes of antimicrobial or antibiotic drugs have been generated, but the antimicrobial resistance issue is far of being solved. The most important discoveries are summarized in Table 2 [1,3,6]. Other antimicrobial agents [7] include: (i) *disinfectants* such as bleach (NaOCl), hydrogen peroxide, *N*-chloro compounds; (ii) *antiseptics* such as iodine as found in povidone iodine [8,9] and potassium iodide, phenols and alkylhalophenols (hexachlorophene and triclosan, benzoic/salicylic acids, quaternary ammonium salts); and (iii) *preservatives*: NaCl, nisin, mercury-derived compounds, and formaldehyde or its aqueous form, formalin.

**Table 2:** Discovery of antimicrobial drugs during 1930s–2000s.

1930s	1940s	1950s	1960s	1970s	1980s	1990s	2000s
Sulfonamides	Beta-lactams *	Glycopeptides	Lincosamides	Trimethoprim			Oxazolidinones
	Aminoglycosides	Chloramphenicol	Quinolones				Lipopeptides
		Tetracyclines	Streptogramins				
		Macrolides					

* Penicillin was the first beta-lactam discovered. Other beta-lactams were the cephalosporins and carbapenems discovered in 1960s and 1980s respectively. Generated data from [1,3,6] with permission.

Recent studies aim to involve ecologic strategies for treating infections by using natural compounds, as plant extracts and volatile oils [10,11,12]. Since the use of antibiotics and other antimicrobials has become less effective, many times the use of high amounts of active drugs is needed to solve life-threatening infections. Antimicrobials used in high amounts can prove to be toxic for patients, causing severe side effects. Therefore, one of the most desired antimicrobial strategies relies on the use of nanosized carriers to transport and control the release of the active antimicrobial [13]. Recently, due to the AMR to synthetic drugs, there has been a tendency towards plant-derived agents that exhibit antimicrobial properties such as the use of essential oils [10,14,15]. Despite their proven antimicrobial effects, the use of essential oils is still limited in anti-infectious therapy due to the low stability and high volatility of the active compounds. For this reason, transport vehicles with nanosized dimensions are also required to achieve the best results of this type of therapy [16].

In the current work we do not consider which of the above mentioned antimicrobial drugs is more effective, but rather trying to review the impact of incorporating them into magnetic nanoparticles to enhance the efficacy of the currently used natural and synthetic antimicrobials by acting as efficient carriers allowing them to better reach the specific sites and improving their properties. 

## 2. Antimicrobial Approaches Based on Nanotechnology

Nanoparticles (NPs) in general have many properties that are different from those of traditionally used materials [10,17,18,19,20,21,22,23,24,25,26,27,28]. They have dimensions typically below 100 nm, which allows them to reach specific sites inside the body and even to be permeable to tissues and cells. Therefore, they can deliver the drugs in active forms at sites that conventional drugs may not reach by themselves and thus minimise the undesirable side effects [29]. Additionally, they have high surface to volume ratio, therefore by using a small amount of drug, the exposure of it at the point of interest will be maximized and thus any toxicity issues of the incorporated drug can be avoided [29,30,31]. Moreover, by integrating different coatings with drugs, non-acute and time controlled drug delivery can be achieved. This is very important for treating infectious diseases since the delivery of the drug should be controlled both in time and in quantity.

Many nanosized structures can be used as drug carriers such as liposomes, synthetic or natural polymers, inorganic and metallic NPs, dendrimers, silicon and others. Iron oxide magnetic NPs (MNPs) have many advantages and are considered very promising drug carriers. They can be handled by using an external magnetic field and thus directed to a specific area in the body where they then excrete the medicine, avoiding delivery to unwanted tissues. MNPs can be formed either from pure metals (cobalt, nickel, manganese and iron) or alloys and their oxides [32]. However, only the iron oxide MNPs have been approved for clinical use by the Food and Drug Administration due to their inherent properties [29]. They are biocompatible since iron oxides-magnetite and maghemite-occur naturally in the human heart, spleen and liver. This makes them non-toxic and biocompatible at physiological concentrations, where an upper limit of MNPs for biomedical applications should be estimated [33]. Other advantages of iron oxide MNPs are the ease of producing them from alkaline co-precipitation of Fe^2+^ and Fe^3+^, their chemical stability under physiological conditions and their facile chemical modification by using various shells such as gold, polymers, silanes, dendrimers and others [29,34].

## 3. Magnetite Nanoparticles Used in Biomedical Applications

Iron oxide MNPs have been used with various synthetic and natural drugs to inhibit the development of fungi, yeasts and bacteria that can cause serious infectious diseases. They have potential advantages due to their size, the low amount of drug needed to inhibit microbial multiplication, the option to image them and their manipulation using magnetic fields [11,35]. Additionally, Fe_3_O_4_ MNPs have been used recently for antigen or vaccine delivery and respiratory diseases. Vaccines have been used for the protection and control of a range of infectious diseases such as malaria, acquired immunodeficiency syndrome (AIDS) and others. The development of new vaccine formulations that will transfer the active substances in a favorable way is very important. MNPs in the viral size have high intracellular uptake, whereas CD4 and CD8T cells and antibodies could be elicited when antigen is incorporated on the NPs surfaces. These properties of MNPs make them advantageous to previous applied methods [36]. The advantages of MNPs for treating respiratory diseases relay on the fact that nanoaerosols carriers can reduce the amount of the drug deposited into the lungs, avoid biological metabolism and deposition in the liver, penetrate into the lungs and reach alveoli due to their small size, target specific tissues and also having the possibility of being imaged using nano-optical techniques [37].

## 4. Functionalized Magnetite Nanoparticles to Treat Infections

**Figure 2:**
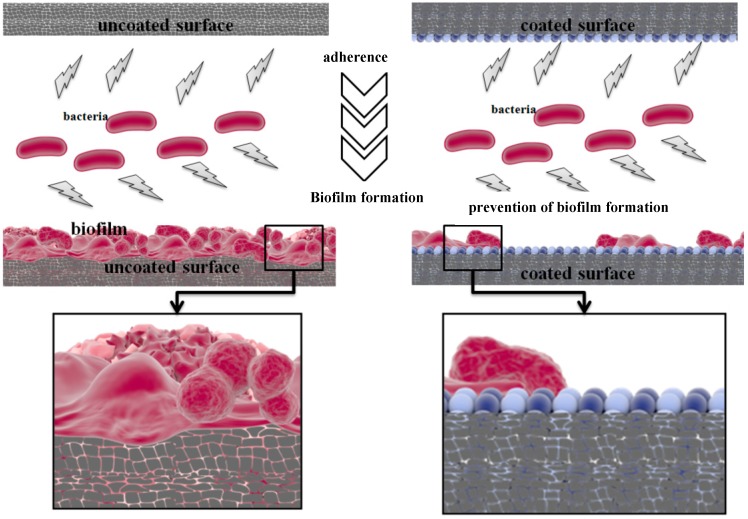
Schematic representation of microbial biofilm development in the presence and absence of functionalized bioactive magnetite nanoparticles.

In recent years, researchers valorized the potential of magnetite nanoparticles to be used in the inhibition of microbial growth and biofilm formation of many pathogenic species, such as fungi, yeasts and bacteria and also to treat specific infectious diseases. The microbial biofilm inhibition with the usage of MNPs is illustrated better in Figure 2, where uncoated surfaces favor the biofilm formation in contrast to the coated surfaces where microbial microorganisms cannot adhere.

### 4.1. MNPs to Inhibit Yeasts

#### 4.1.1. *Candida albicans*

Studies have reported the production of functionalized magnetite nanoparticles with 20 nm maximum diameter, using a precipitation method, with oleic acid as surfactant, and *Rosmarinus officinalis* essential oil (NPs-EO) as an antimicrobial agent [38]. The suspended core-shell nanoparticles were used to coat a catheter by applying a magnetic field on the nanofluid, whereas the essential oil was adsorbed in a secondary covering treatment. The fungal adherence ability on the catheter was examined in multiwell plates, where catheters had been places with and without NPs-EO nanobiosystems, using a culture based evaluation method and confocal laser scanning microscopy (CLSM). Authors reported that the main observed components of *R. officinalis* were 40.596% eucalyptol, 11.389% camphor, 10.19% caryophyllene, and 18.42% α-pinene. The catheter coated with NPs-EO showed a distinct *C. albicans* biofilm inhibitory effect in a time dependent manner, for up to 72 h, as the viable cell counts (VCCs) revealed. *C. albicans* biofilms grown on the nanomodified surfaces diminished with up to approximately 85% as compared to uncoated catheters, as the authors observed using CLSM.

Nanocomposites of 7–17 nm average diameter consisting of biogenic magnetite, silver NPs and chitosan proved to have considerable antimicrobial effects against *C. albicans* [39]. The MIC values of such nanocomposites, depending on the silver content, were in the range of 0.9 to 12.6 mg/L. 

Nystatin, a wide used antifungal drug, was successfully attached to chitosan coated MNPs [40]. The resulting nystatin chitosan-MNPs, with an average diameter of 8 nm, present an enhanced antifungal activity against *C. albicans*. 

#### 4.1.2. *Candida tropicalis*

The functionalized magnetite NPs with *R. officinalis* essential oil coated onto a catheter previously discussed in the *C. albicans* section, also showed a distinct inhibition compared to uncoated catheter against *C. tropicalis* [38]. The lowest number of counted viable cells was observed after 24 h of cell culture, whereas at 48 and 72 h the number of the VCCs was higher; most probably due to the early production of pseudohyphae and germ tubes. Such antimicrobial agents of essential oils into MNPs can be used as biofilms of prosthetic devices against pathogenic microorganisms but also as nanocarriers for treating infectious diseases [39].

#### 4.1.3. *Candida krusei*

MNPs have great potential in the treatment of *Candida krusei* infections, even though at this moment the mechanism of action is unclear. Essential oils have been stabilized with MNPs and used successfully in the prevention of fungal biofilms. In particular, a core/shell/coated-shell hybrid nanobioactive system composed from *Anethum graveolens* essential oil-magnetic nanoparticles was obtained by a modified Massart method. GC-MS of the *A. graveolens* essential oil revealed that the main components were limonene (56.53%) and carvone (39.56%). CLSM on coated and uncoated coverslips colonized with *C. krussei Y5* strain revealed a rare adherence of yeast cells on the coated surface, whereas a thin biofilm with an internal canalicular structure was developed on the uncoated surface [12]. Other recent study reports the modulation of *C. krusei* infections by MNPs [18]. This modulation is dependent by the surface of MNPs and the authors pointed out that microbial interactions are influenced by interfacial forces, such as the electrostatic field, that plays a significant role on the interaction between MNPs and microbial surfaces [41].

#### 4.1.4. *Candida glabrata*

Similarly, the *A. graveolens* essential oil-MNPs nanoparticles were used to prevent the growth of *C. glabrata*. Studies revealed that *C. glabrata* inoculated coupons produce a monostratified biofilm, homogenously distributed on the uncoated coverslip surface as well as on the coated one, in which a central large microbial colony exhibited a midpoint disruption [12]. This midpoint disruption revealed an inhibition mechanism of the MNPs. More studies are needed to prove the degree of the antimicrobial resistance in response to the essential oil concentration with these microorganisms. 

#### 4.1.5. *Saccharomyces cerevisiae*

Recently, fatty acid-functionalized magnetite nanostructures were used to exemplify a series of available methods for the investigation of *in vitro* microbial biofilms developed on different substrata, using as experimental model a *S. cerevisiae* strain. The results are very promising, highlighting the importance of magnetic nanoparticles in the inhibition of fungal biofilms. The CLSM technique was used to obtain images of uncovered and nanoparticles-oleic acid covered glass-slips and the growth of *S. cerevisiae* was monitored after 24, 48 and 72 h. Authors have demonstrated that the cover slips coated with oleic acid-MNPs showed higher microbial colonization inhibition compared to the uncoated surfaces [42]. 

The above yeasts have many implications and are present in many respiratory or pulmonary diseases and therefore the usage of essential oils could be effective in treating such infectious diseases. Nevertheless more essential oils, other than *A. graveolens* have to be tested with these yeasts to identify those with the best antimicrobial activity. Additionally, GC-MS analysis of such essential oils will provide information on which specific molecules and which concentration is responsible for the specific resistance on biofilm formation.

The most relevant results and the phenotypes altered in yeasts by functionalized MNPs are summarized in Table 3.

**Table 3:** List of MNPs in combination with active compounds to inhibit yeasts.

Type of Nanoparticles	Altered Microbial Phenotype	Bacteria Species	Reference
MNPs/*Rosmarinus officinalis*	Adherence, biofilm formation	*Candida albicans*	[38]
MNPs/Ag/chitosan	Adherence, biofilm formation	*Candida albicans*	[39]
MNPs/chitosan/nystatin	Growth on solid media	*Candida albicans*	[40]
MNPs/*Rosmarinus officinalis*	Adherence, biofilm formation	*Candida tropicalis*	[38]
MNPs/chitosan/nystatin	Growth rate	*Candida tropicalis*	[39]
MNPs/*Anethum graveolens*	Adherence, biofilm formation	*Candida krusei*	[12]
MNPs/dextran	Growth	*Candida krusei*	[41]
MNPs/*Anethum graveolens*	Adherence, biofilm formation	*Candida glabrata*	[12]
MNPs/oleic acids	Adherence, biofilm formation	*Saccharomyces cerevisiae*	[42]
MNPs/Ag/chitosan	Growth	*Candida parapsilosis*	[39]

### 4.2. MNPs to Inhibit Bacteria

#### 4.2.1. *Escherichia coli*

Recent studies have reported magnetite nanoparticles (MNPs) coated with chitosan and grafted with cephalosporins that show great antibacterial properties against *E. coli* [25]. The magnetic chitosan microspheres were prepared by wet chemical precipitation of Fe^2+^ and Fe^3+^ ions in aqueous solution with chitosan and sodium hydroxide. The tested antibiotics were cefepime, ceftriaxone, cefuroxime and cefoperazone. The cephalosporins were well encapsulated into the magnetite chitosan microspheres, retaining their properties and being advantageous to conventional delivery of the drug since by using this magnetite/chitosan approach, the minimal inhibitory concentration was decreased from 2 to 7.8 times for the *E. coli* tested strains. It was also noticed that the drying temperature of the resulting magnetite/chitosan microspheres was very important, since it was found that 100 °C resulted in MICs approximately half value as compared with the situation when the temperature was of 50 °C. Cerufoxime was the exception, were both 50 and 100 °C had similar MICs, but again much lower (0.075 μg/mL) than without the use of microspheres (0.188 μg/mL).

Polyacrylamide doped MNPs (10–20 nm) were reported to display excellent bactericidal properties, especially in the elimination of microbes from water [43]. The MIC of these MNPs on *E.* *coli* was 60 μg/mL and the authors pointed out that the superoxide and hydroxide radicals produced by iron oxide seem to be the cause of bacterial damage, since it may result in oxidative stress, damage of proteins, membranes and DNA [43]. 

Modified MNPs with sodium poly(γ-glutamic acid) (PGA) were found to lower the MIC of the commercial antibiotics linezolid and cefaclor against *E. coli*, as compared with solutions of the plain antibiotics [44]. For the *E. coli* ATCC 8739 strain, the PGA-coated MNPs showed a lower MIC value (<0.5 μg/mL) than linezolid (16 μg/mL) and cefaclor (8 μg/mL), but higher MIC values for the CaPGA-coated MNPs (128 μg/mL). It was found that the coated PGA-MNPs do not reduce the MIC values for the *E. coli* O157:H7 strain compared to commercial antibiotics, but nevertheless they showed a certain inhibition against all tested strains. In another work, MNPs stabilised with thioglycerol showed effective inhibition against *E. coli* [45]. Such stabilised iron oxide nanoparticles have potential applications in the biomedical field, mainly as antimicrobial agents.

Dextran and sucrose coated MNPs (with diameter of 5.8 and 7.3 nm respectively) showed good inhibition against *E. coli* ATCC 25922 strain, especially at concentrations between 0.01–0.625 mg/mL [41]. This study also revealed that dextran is more effective as a coating medium regarding bactericidal activity compared to sucrose. The authors demonstrated in the same study that parameters such as hydroxyl compounds, active oxygen generated species, reduced size of nanoparticles and usage of specific sugars for the microbial enzymatic conversion play significant roles in the bactericidal efficacy and mechanism. 

#### 4.2.2. *Staphylococcus aureus*

Magnetite NPs cross-linked with chitosan produced by the co-precipitation method and grafted with two selected aminoglycoside antibiotics (kanamycin and neomycin) were also reported [27]. The synthesised chitosan-magnetite NPs coated with aminoglycosides proved to possess exceptional antibiotic activity against *S. aureus* strains. The amount of both kanamycin and neomycin used in the magnetite NPs was significantly less than the one without NPs that was necessary to stop the growth of *S. aureus*. As an approximation the amount of kanamycin or neomycin used with chitosan-magnetite NPs needed to stop the growth of *S. aureus* was half the amount of these antibiotics required without MNPs. The reason of this exceptional antimicrobial activity was the higher surface area to volume ratio of the MNPs and hence the greater available surface of the antibiotic that was in contact with the microorganisms but also the control release ratio. The magnetic chitosan microspheres mentioned in the *E. coli* section [25] retained their properties and were advantageous for conventional delivery of the drug since by using this magnetite/chitosan approach the minimal inhibitory concentration was decreased from 2 to 7.8 times for the *S. aureus* strains. The drying temperature of the resulting magnetite/chitosan microspheres was very important, since it was found that 50 °C did not affect the efficiency of the drug, but 100 °C enhanced the activity of cephalosporins by reducing the MIC from 2 to 7.8 times as mentioned before. In this work by Chifiriuc *et al.* it was also mentioned that chitosan had a positive effect on the antimicrobial activity by encapsulating well the cephalosporins, making them available for interacting with the bacterial microorganisms.

Recently, Grumezescu *et al.* [46] found that spherical magnetites containing eugenol and prepared by the precipitation method had very good anti-adherence activity against *S. aureus* biofilm formation. To prepare such NPs, 3-hydroxybutyric acid-co-3-hydroxyvaleric acid, polyvinyl alcohol and eugenol were used as the organic phase to prepare the emulsion, which after sonication, water addition, chloroform evaporation and centrifugation gave the magnetites. The MNPs with eugenol were fabricated by matrix assisted pulsed laser evaporation (MAPLE). The size of the MNPs was less than 10 nm and the microbiology resulted demonstrated that such MNPs with eugenol had very good anti-biofilm activity against *S. aureus*.

MNPs coated with chitosan-carboxymethylcellulose were found to have improved antibiotic activity when incorporated with known antibiotics [26]. Such Fe_3_O_4_/chitosan-carboxymethylcellulose MNPs were found to enhance considerably (2%–10%) the efficacy and drug delivery of penicillins, macrolides, aminoglycosides, rifampicines and quinolones classes against *S. aureus*. Thus, such MNPs can be used as potential carriers of antibiotics by enhancing their effectiveness without being cytotoxic or influencing the HCT8 eukaryotic cell cycle.

#### 4.2.3. *Pseudomonas aeruginosa*

Magnetite NPs cross-linked with chitosan and coated with aminoglycoside antibiotics (kanamycin and neomycin) were also used to stop the growth of *P. aeruginosa* [27]. The incorporation of the NPs with these antibiotics clearly enhanced the antimicrobial activity of the last due to the higher surface area to volume ratio and to the controlled release of the aminoglycosides. MNPs functionalized with eugenol had also similar anti-adherence activity against *P. aeruginosa* strains [46], making them ideal candidates for developing new anti-bacterial materials. Effective against *P. aeruginosa* were also the MNPs coated with chitosan-carboxymethylcellulose and incorporated with antibiotics [26]. Nanocomposites consisting of biogenic magnetite, silver NPs and chitosan exhibited MIC values between 14 to 50 mg/L against *P. aeruginosa*, lower than the plain drugs [39]. MNPs stabilized with thioglycerol showed also a good inhibition against *P. aeruginosa* with a MIC value of 0.047 mg/mL [45].

#### 4.2.4. *Enterococcus faecalis*

Chifiriuc *et al.*, reported the first study trying to investigate the ability of magnetite nanoparticles to improve the anti-bacterial activity of current antibiotics against planktonic and biofilm-growing *E. faecalis*. The antibiotics tested with MNPs were vancomycin, penicillin and streptomycin. The results suggested that magnetite nanoparticles can be considered effective aminoglycoside antibiotics carriers, in order to obtain improved strategies for elimination of *E. faecalis* biofilms on biomedical devices or human tissues. Also, it was found that MNPs improved the antimicrobial activity of streptomycin, both against planktonic and different *E. faecalis* cells most probably due to the binding effect of MNPs to bacteria and the consequent membrane disruption. The authors pointed out that since this is the first work with the use of MNPs for the elimination of *E. faecalis* from prosthetic devices or human tissues, more work is needed with the use of a variety of antibiotics to understand better the inhibition mechanisms [47].

The most recent findings regarding the impact of MNPs on different bacteria species and the influenced phenotypes are briefly represented in Table 4.

**Table 4:** List of plain and functionalized MNPs derivatives with effect on several microbial phenotypes.

Type of Nanoparticles		Altered Microbial Phenotype		Bacteria Species		Reference	
MNPs/chitosan/cephalosporins		Viability, growth		*Escherichia coli*		[25]	
MNPs/Ag/chitosan		Viability, growth		*Escherichia coli*		[39]	
Polyacrylamide doped MNPs		Viability		*Escherichia coli*		[43]	
MNPs/Na-PGA		Viability, growth		*Escherichia coli*		[44]	
MNPs/Ca-PGA							
MNPs stabilized with thioglycerol		Viability, growth		*Escherichia coli*		[45]	
66 nm of iron oxide MNPs		Viability, growth Inhibition on solid media		*Escherichia coli*		[48]	
Dextran coated MNPs		Viability, growth		*Escherichia coli*		[41]	
sucrose coated MNPs							
MNPs/chitosa/aminoglycosides		Growth		*Staphylococcus aureus*		[27]	
MNPs/chitosan/Cephalosporins		Viability, growth		*Staphylococcus aureus*		[25]	
Spherical MNPs coated with eugenol		Adherence, biofilm formation		*Staphylococcus aureus*		[46]	
MNPs/chitosan/ antibiotics (penicillins, macrolides, aminoglycosides, rifampicines quinolones)		Viability, growth		*Staphylococcus aureus*		[26]	
MNPs/Ag/chitosan		Viability, growth		*Staphylococcus aureus*		[39]	
MNPs stabilized with thioglycerol		Viability, growth		*Staphylococcus aureus*		[45]	
66 nm of iron oxide MNPs		Viability, growth		*Staphylococcus aureus*		[48]	
MNPs/chitosan/ aminoglycosides (kanamycin and neomycin)		Viability, growth		*Pseudomonas aeruginosa*		[27]	
Spherical MNPs coated with eugenol		Adherence, biofilm formation		*Pseudomonas aeruginosa*		[46]	
MNPs/chitosan/carboxymethylcellulose/antibiotics (penicillins, macrolides, aminoglycosides, rifampicines quinolones)		Viability, growth		*Pseudomonas aeruginosa*		[26]	
MNPs/Ag/chitosan		Viability		*Pseudomonas aeruginosa*		[39]	
MNPs stabilized with thioglycerol		Viability, growth		*Pseudomonas aeruginosa*		[45]	
MNPs coated with vancomycin, penicillin and streptomycin		Adherence, biofilm formation		*Enterococcus faecalis*		[47]	
MNPs/Ag/chitosan		Viability, growth		*Enterococcus faecalis*		[39]	
dextran coated MNPs		Viability, growth		*Enterococcus faecalis*		[41]	
sucrose coated MNPs							
MNPs/Ag/chitosan		Viability, growth		*Staphylococcus epidermidis*		[39]	
66 nm of iron oxide MNPs		Growth inhibition on solid media		*Staphylococcus epidermidis*		[48]	
MNPs/Ca-PGA		Viability, growth		*Salmonella enteritidis*		[44]	
MNPs/Na-PGA							
MNPs/Ag/chitosan		Viability, growth		*Klebsiella pneumoniae*		[39]	
MNPs coated with amino acids (l-arginine and l-lysine)		Viability, growth		*Listeria monocytogenes*		[49]	
MNPs stabilized with thioglycerol		Viability, growth		*Bacilus subtilis*		[45]	
66 nm of iron oxide MNPs		Viability, growth		*Bacilus subtilis*		[48]	
66 nm of iron oxide MNPs		Viability, growth		*Bacilus licheniformis*		[48]	
66 nm of iron oxide MNPs		Viability, growth		*Bacilus brevis*		[48]	
66 nm of iron oxide MNPs		Viability, growth		*Vibrio cholerae*		[48]	
66 nm of iron oxide MNPs		Viability, growth		*Streptococcus aureus*		[48]	

**Figure 3:**
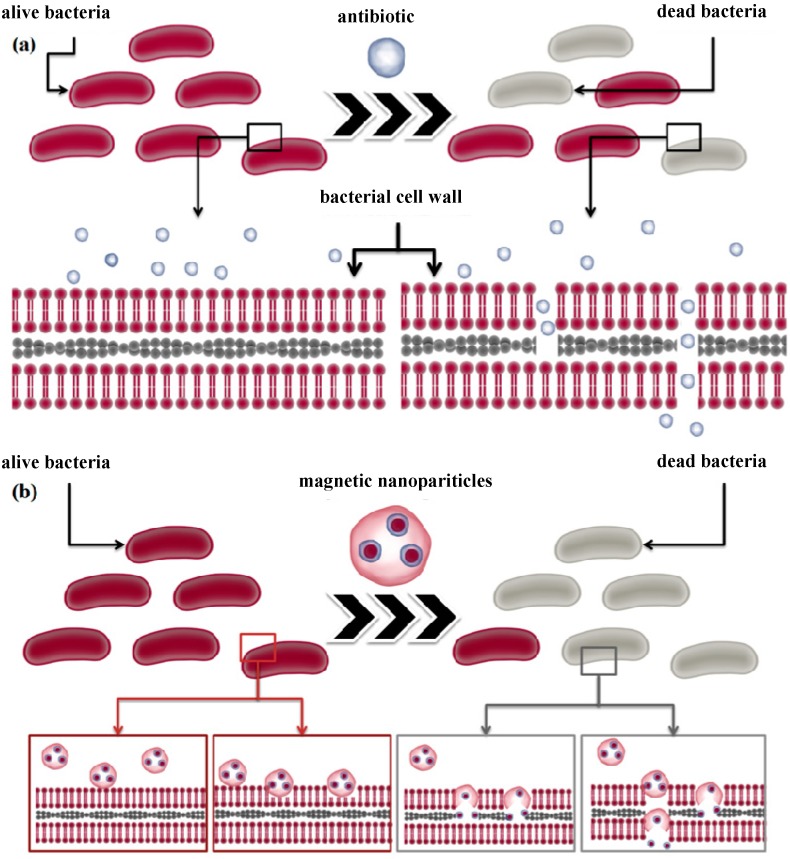
Schematic representation of the possible interaction between bacteria with (**a**) antibiotics and (**b**) magnetic nanoparticles - antibiotics nano-carriers.

An example of the mechanism by which MNPs functionalized with antibiotics improve the antimicrobial inhibition is illustrated in Figure 3. Like the antibiotics which act by interrupting the cell wall synthesis (Figure 3a), studies have revealed that magnetite nanoparticles coated with a chitosan polymeric matrix are able to also bind to bacterial cell walls and interrupt its synthesis. Due to their positive charge these magnetic nanoparticles loaded with antimicrobials have the ability to bind to the bacteria cell surface and alter the cell wall integrity [25], delivering the drug to its target more efficiently (Figure 3b). Due to the limited research on the field, there is not much information on the specific mechanism of interaction between magnetite nanoparticles and bacteria cells. Therefore, the proposed mechanism is putative, and is based on other findings highlighting the nanoparticles-bacteria cell interaction in the case of other nanosized structures such as silver [50,51]. 

### 4.3. MNPs to Treat Respiratory Diseases

Superparamagnetic iron oxide NPs (SPIONs) entrapped into aerosol droplets have been recently studied using a theoretical model but also an *in vivo* mice model to target infected areas of lungs and to deliver such medical droplets. These NPs are so-called nanomagnetosols. A magnetic field was used to direct and target the SPIONS medical droplets into the lungs. With this method, a specific and controlled drug delivery process can be achieved that can be beneficial to traditional aerosol therapies. The authors suggested that these results can be promising for treating localised diseases in lung, for targeting areas of bacterial infections and tumor nodules [52].

Recently, an aerosol system was designed with biological substances and the usage of MNPs for enhancing the active substances delivery into the lungs. The MNPs of 65 nm size were synthesized using the co-precipitation method of ferric and ferrous chloride salts with ammonia and dextran as a coating. The suspension of MNPs was added to 1% of glucose solution for the evaluation of the delivered dose. Antibiotics were also used such as kanamycin and streptomycin and their delivery using MNPs was tested. The device was capable of producing nanoaerosols of 10–300 nm size with concentration up to 2 × 10^7^ cm^3^ and it was demonstrated that approximately 0.4 μg of it was delivered in 1 h into a mouse lung, using its nose as the exposure diode. The authors described that such nanoaerosol generators could be used for treating infectious lung diseases and other respiratory related infections [37]. 

## 5. Conclusions and Perspectives

As the microbial resistance problem continues to mount, alternative preventive and therapeutic approaches are emerging. Magnetic nanoparticles have proved to be a very interesting and useful tool in handling infections, contributing significantly to the delivery of antibiotics or other active antimicrobial agents to specific sites in the human tissue. Their small sizes, good biocompatibility, and biochemical properties as well the great antimicrobial effect recommend these nanobioactive magnetic nanostructures as successful candidates for the development of future antimicrobials. Such encapsulated MNPs with antimicrobial agents can be used for the coating of medical or prosthetic devices, as nanocarriers for the delivery of specific and time-eluting drugs to specific tissues, and can minimize toxicity effects due to the small amount of antibiotic that they carry. The recent research orientation towards plant-derived pharmaceutical agents makes volatile oils very promising and potential candidates to treat infectious diseases, ranging from simple ones, such as used for producing antimicrobial hands cream, to more advanced ones, such as nano-aerosols for treating respiratory infections. The usage of MNPs proved to be very important for the stabilization and for the efficient targeted delivery of many antimicrobial agents. Parameters such as size of the MNPs, concentration of active drugs and polymers, type of coatings or stabilizing agents and drug delivery time represent important factors for the treatment of infectious diseases and more research is needed to understand the intimate mechanisms of a particular effect against certain microorganisms such as the bacteria and fungi with high AMR. Nevertheless, the results discussed in this review are promising, showing that MNPs—antimicrobial systems can be advantageous compared to classical, plain antimicrobials but there are still open questions to understand better the mechanisms of microbial inhibition and to test various antimicrobial agents in different nanocarrier systems in numerous microorganisms that can provoke challenging infectious diseases. 
